# Model Based on an Effective Material-Removal Rate to Evaluate Specific Energy Consumption in Grinding

**DOI:** 10.3390/ma12060939

**Published:** 2019-03-21

**Authors:** Amelia Nápoles Alberro, Hernán A. González Rojas, Antonio J. Sánchez Egea, Saqib Hameed, Reyna M. Peña Aguilar

**Affiliations:** 1 Department of Mechanical Engineering (EPSEVG), Universidad Politécnica de Cataluña, Av. de Víctor Balaguer, 1, Vilanova i la Geltrú, 08800 Barcelona, Spain; amelia.napoles@upc.edu (A.N.A.); hernan.gonzalez@upc.edu (H.A.G.R.); hameeds@tcd.ie (S.H.); 2 Department of Mechanical Engineering (EEBE), Universidad Politécnica de Cataluña, Av. Eduard Maristany, 16, 08019 Barcelona, Spain; 3 Department of Fluid Mechanics (EEBE), Universidad Politécnica de Cataluña, Av. Eduard Maristany, 16, 08019 Barcelona, Spain; reyna.mercedes.pena@upc.edu

**Keywords:** power consumption, material-removal rate, specific energy consumption, grain density, modeling

## Abstract

Grinding energy efficiency depends on the appropriate selection of cutting conditions, grinding wheel, and workpiece material. Additionally, the estimation of specific energy consumption is a good indicator to control the consumed energy during the grinding process. Consequently, this study develops a model of material-removal rate to estimate specific energy consumption based on the measurement of active power consumed in a plane surface grinding of C45K with different thermal treatments and AISI 304. This model identifies and evaluates the dissipated power by sliding, ploughing, and chip formation in an industrial-scale grinding process. Furthermore, the instantaneous positions of abrasive grains during cutting are described to study the material-removal rate. The estimation of specific chip-formation energy is similar to that described by other authors on a laboratory scale, which allows to validate the model and experiments. Finally, the results show that the energy consumed by sliding is the main mechanism of energy dissipation in an industrial-scale grinding process, where it is denoted that sliding energy by volume unity decreases as the depth of cut and the speed of the workpiece increase.

## 1. Introduction

Efficiency in machining processes requires more attention due to the high cost of energy, in which the manufacturing cost represents a significant proportion of the total cost of the final product [1]. The Industry 4.0 philosophy presents a global vision of virtualization for manufacturing high-quality parts [2,3]. These models and simulations help to optimize the conditions to execute the work cycle and desired results in manufacturing parts [4,5]. Hence, it is deduced that both energy efficiency and virtualization require a model to analyze the behavior of the different manufacturing processes with respect to operating conditions.

The models of specific energy are divided into two main groups: the models that evaluate specific cutting energy SCE, and the models that calculate specific energy consumption SEC. The first group is based on experimental measurements of cutting forces during machining by using a piezoelectric dynamometer located on the table of the grinding machine [6] or in the spindle where the grinding wheel is attached [7]. In this case, the recorded values of forces are multiplied with the peripheral speed of the grinding wheel to define cutting-power consumption. Other authors estimated SCE by developing a function that related the active power of motor with the mechanical power developed by the spindle during turning [8]. The same strategy was also used by González et al. [9] in drilling to investigate the influence of different cutting conditions.

However, the second group evaluated SEC during the process by measuring the active power of the motor, as was done by Diaz et al. [10] during milling, or Sánchez Egea et al. [11] during turning operations. Moreover, there are two ways to obtain material-removal rate $Q_{w}$. The first is defined as the product of the cutting cross section and workpiece speed. The other is defined as the product of the effective section of cutting grains and the cutting speed of the grinding wheel [12]. Generally, the authors used the first model, in which they considered the cutting section by the depth of cut and width of the grinded zone [13]. Conversely, in the second model, the authors considered the effective cross section of the cutting chip and number of grains corresponding to the contact area [14]. To make the second model applicable, the researchers used an equation for the maximum thickness of the undeformed chip [15]. Based on the geometrical characteristics of chip formation, this thickness is defined as a function of cutting speed, workpiece speed, depth of cut, and the diameter of the grinding wheel. This equation also included the normalized density of static grains $C_{g}$ and a constant that indicated the average grain geometry. There are cases in which $C_{g}$ is defined as a function of angle of attack of the grain [16]. Normally, the authors calculated chip thickness by using the empirical data of $C_{g}$ [17]. Due to the complexity of the cutting edges of the grinding wheel, it is well known that $C_{g}$ significantly influenced grinding behavior. Therefore, several authors measured the topography of a grinding wheel through an electron microscope [18]. Unlike the classic methods of estimating $Q_{w}$, Nadolny [19] proposed a new index $SI_{Q}$ that defines the material-removal rate of a single abrasive grain, which is based on the number of active kinematic cutting grains. So far, SEC models are characterized by the macro level during iteration between workpiece and grinding wheel to predict the average value of chip thickness. A recent work developed the model of normal and tangential forces by considering the microinteraction between workpiece and grinding tool [20].

In the present work, SEC is obtained by measuring the active power consumed by the motor that drives the grinding wheel. A model was developed to calculate the $Q_{w}$ under different cutting configurations and taking into account the interaction between grains and workpiece. Active power consumption is measured by a power analyzer connected to the three-phase electric motor. An equation of deformed-chip thickness and effective cutting section is also proposed to accurately define the material-removal rate. Finally, the chip-thickness equation is defined as a function of the radial position of each grain, cutting parameters, and actual grain density of the grinding wheel. Additionally, a laser distance sensor was used to measure the topography of the abrasive wheel and, ultimately, to calculate grain density.

## 2. Model of Specific Energy Consumption

In grinding, three mechanisms occurred between grinding wheel and workpiece. First, the friction between wheel and workpiece, characterized by negligible small $Q_{w}$. When the force of grains increased on the workpiece, elastic and plastic deformation occurred, which produced a scratch with crests on the sides. The material was removed by increasing the force to produce chip formation [21]. In this work, it was considered that the consumed power in the grinding is due to the power dissipated by different mechanisms involved in the process. These mechanisms were the friction between wheel and workpiece (sliding), plastic deformation without breakage (ploughing), and chip removal by shearing (chip formation) [22]. The power consumed by the sliding, ploughing, and chip-formation mechanisms are $P_{sl}$, $P_{pl}$ and $P_{ch}$, respectively. Then, total consumed power *P* during the process is equal to the sum of power consumed by each of the above-mentioned mechanisms.

(1) $$P=P_{sl}+P_{pl}+P_{ch}$$

In the chip-removal process, SEC is directly proportional to the relation between consumed power and $Q_{w}$ [23]. If Equation (1) is divided by $Q_{w}$ and reorganized, then Equation (2) is obtained as follows:

(2) $$\frac{P-P_{sl}}{Q_{w}}=\frac{P_{pl}}{Q_{w}}+SEC_{ch}$$

where $SEC_{ch}$ is the specific energy consumed by chip formation.

During grinding, two types of cutting operations were defined due to the alternative movement of the table on which the workpiece was placed. If the movement was in the opposite direction to peripheral speed $v_{c}$ of the grinding wheel, then this operation is called up-grinding. There are three associated mechanisms here: sliding, ploughing, and chip formation. If the movement is in the same direction as the $v_{c}$ of the wheel, then the operation is called down-grinding. In this particular case, there is only one mechanism associated, which is sliding. In this work, grinding was conceived in the following way: the depth of cut was applied to the workpiece when it started its movement in up-grinding. This step should not be repeated until down-grinding is completed. Therefore, during the up-grinding movement, sliding, ploughing, and chip formation existed simultaneously. On the other hand, during down-grinding, only sliding was expected. Accordingly, the power consumed during up-grinding and the power consumed during down-grinding are known, so then the mechanisms of ploughing and chip formation can be isolated. Hence, the difference between the power consumed in up- and down-grinding was due to ploughing and chip formation, which were the mechanisms that characterized cutting [24]. In this study, the power of the two trajectories was measured by a power analyzer during grinding in a dry condition with different cutting conditions and metallic alloys. The power consumed by the motor was also measured during an idle condition, i.e., when the wheel was not in contact with the workpiece. Therefore, active power consumption can be calculated by subtracting the power measured without cutting (idle) from the power in up- and down-grinding.

### Model of Effective Material-Removal Rate in Grinding

**Figure 1:**
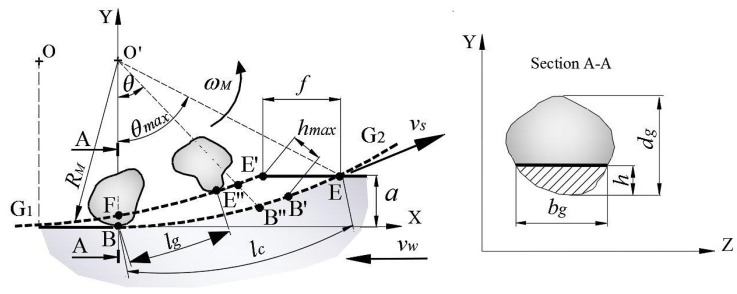
Characteristics of interaction between grinding wheel and workpiece.

In grinding, it is difficult to define the geometry of the cutting tool, as the grinding wheel has different cutting grains, distributed irregularly on the working surface and, at the same time, grains have different cutting edges. The material-removal rate is obtained by considering the geometric intersections between grinding wheel and workpiece, as well as the multiple grains involved in cutting. To define the model of material-removal rate $Q_{w}$, the equation of chip thickness and the section cut by a grain $A_{cg}$ was first obtained. Subsequently, the $Q_{w}$ by all cutting grains is simultaneously calculated. Figure 1 represents the section of the removed material during up-grinding. It defined the radius of grinding wheel $R_{M}$, angular position of grain θ, contact length between grinding wheel and workpiece $l_{c}$, and cutting parameters such as speed of grinding wheel $v_{c}$, speed of workpiece $v_{w}$, and depth of cut *a*. Undeformed chip thickness *h* was measured in the XY plane, and $A_{cg}$ was evaluated in the ZY plane, which is perpendicular to the plane of the grinding wheel and is represented by the A-A section.

In this section, the evolution of chip thickness as a function of angular position of grain θ was analyzed. To define chip thickness, it was assumed that the grains of the grinding wheel were equally spaced, like the teeth of a milling cutter. Accordingly, Figure 1 shows the trajectories $G_{1}$ and $G_{2}$ of two abrasive grains that were consecutively cut. Trajectory $G_{2}$ has a center displaced at a distance $OO^{\prime }$, equivalent to feed rate *f* that depends on the distance between grains $l_{g}$, and speeds of workpiece $v_{w}$ and grinding wheel $v_{c}$. The zone of interest was defined by points $BEE^{\prime }F$, where the $l_{g}$ between grinding wheel and workpiece was defined by arc BE, and maximum thickness by points $E^{\prime }B^{\prime }$. To obtain the coordinates of the intersection of line $E^{\prime \prime }B^{\prime \prime }$ with curves $G_{1}$ and $G_{2}$, equations were developed to define the circumferential arcs of $G_{1}$ and $G_{2}$ and line $O^{\prime }B^{\prime \prime }$. Then, point $E^{\prime \prime }$ was defined by the intersection of curve $G_{1}$ and line $O^{\prime }E^{\prime \prime }$ as a function of θ. Therefore, the equations can be defined as a function of dimensionless angular position $\theta^{*}$ defined as the ratio of θ and $\theta_{max}$.

(3) $$\theta^{*}=\frac{\theta }{\sqrt{2\cdot a/R_{M}}}$$

Chip thickness was defined as:

(4) $$e=2\theta^{*}\cdot \left(l_{g}\cdot \frac{v_{w}}{v_{c}}\right)\cdot {\left(\frac{a}{D_{M}}\right)}^{1/2}$$

where $D_{M}$ is the diameter of the grinding wheel.

By considering the static density of the grain constant, it was estimated that the distance between grains is constant throughout the perimeter of the grinding wheel. Then length between grains $l_{g}$ can be deduced as:

(5) $$l_{g}=\frac{1}{C_{g}\cdot b_{g}}$$

where $b_{g}$ is the width of grain as a function of undeformed chip thickness *h* and diameter of grain $d_{g}$.

(6) $$b_{g}=2\cdot \sqrt{d_{g}\cdot h}$$

Replacing Equations (5) and (6) in Equation (4) gave a useful expression for *h*, as follows:

(7) $$h={\left(\frac{\theta^{*}\cdot v_{w}}{C_{g}\cdot v_{c}}\right)}^{2/3}\cdot {\left(\frac{a}{d_{g}\cdot D_{M}}\right)}^{1/3}$$

The area of the material removed by grain $A_{cg}$ corresponded to the effective section of cutting by grain. To estimate $A_{cg}$, it was assumed that the geometric shape of grain can be approximated to a sphere, and only a part of the grain cut the material [15]. For a sphere, the effective cutting area is a function of *h* and the radius of grain $R_{g}$:

(8) $$A_{cg}=\arccos\left(1-h/R_{g}\right)\cdot R_{g}^{2}-\left(R_{g}-h\right)\cdot R_{g}\cdot sen\left(\arccos\left(1-h/R_{g}\right)\right)$$

$A_{cg}$ is different for each relative position of the grain as chip thickness e increases with the increase of $\theta^{*}$. The total area of cutting depends on the number of grains and is equal to the sum of instantaneous areas of each grain present along the contact length between wheel and workpiece. Finally, considering number of grains $N_{g}$ cut in the grinding width, material-removal rate $Q_{w}$ by all grains in the ZY plane is calculated as:

(9) $$Q_{w}=\sum_{i=1}^{N_{g}}\left(\vec{A_{cg}}\left(h_{i}\right)\times \vec{v_{c}}\right)$$

## 3. Experiment Setup

In this work, two types of experiments were performed. The topography of the grinding wheel was evaluated and the power consumed by motor was measured during grinding test. Two types of metallic alloys were selected, ductile and brittle. This helps to understand the effect of material hardness and cutting parameters on the SEC.

### 3.1. Estimation of Grain Number Per Unit Area in Grinding Wheel

**Figure 2:**
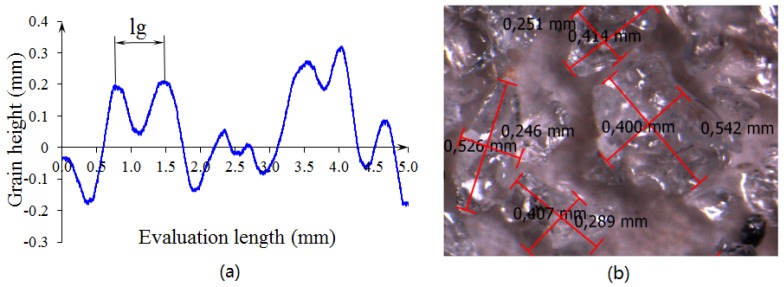
Characteristics of grinding wheel A36H5V: (**a**) surface-roughness profile and (**b**) size of detached grains.

The distance between two adjacent grains depends on the structure of grinding wheel. In grinding tests, the grinding wheel of aluminium oxide A36H5V was used, which has an outside diameter of 250 mm, a 76 mm mounting hole, 40 mm width, and grain-size number 36 according to the manufacturer’s certificate. According to the FEPA standard [25], the characteristics of this grinding wheel are $d_{g}$ = 0.337 mm and $l_{g}$ = 0.67 mm. The topography of the wheel was measured to confirm the information provided by the manufacturer. The wheel was mounted on a divider head located on the table of a vertical milling machine. Measurements were made by using a laser (LDS-Laser distance sensor, model: LDS90/40, LMI Technologies Inc., Burnaby, BC, Canada) located on the spindle of the machine. Surface roughness was measured with an accuracy of 0.001 mm according to the data-acquisition equipment (HBM, model: Spider-8, Hottinger Baldwin Messtechnik GmbH, Darmstadt, Germany). In total, eight profiles of surface roughness, with an evaluation length of 5 mm each, were measured across the width of the grinding wheel. For statistical analysis, the average length value between grains was calculated. The Anderson Darling test was applied to the specimen, and a probability of 0.570 was found. Consequently, it could be assumed that its distribution had normal behavior, as the *p*-value was greater than 0.05. Figure 2a shows the surface-roughness profile of the grinding wheel in which the distance between grains was identified. The average grain number per unit area can be calculated by evaluating the number of peaks in the specimen. The average distance between grains was 0.775 mm, with a confidence interval of 0.650–0.900 mm, and the averaged $C_{g}$ was 3.05 grains/mm${}^{2}$, with a confidence interval of 3.030–3.676 grains/mm${}^{2}$. The value of distance between grains was greater than the theoretical value indicated by the manufacturer and, therefore, the $C_{g}$ was slightly smaller. A single-tip diamond test was also performed with a maximum depth of cut of 0.05 mm and an axial table feed speed of 1.6 mm/s. A total of six tests were performed with a 1.5 carat single-tip diamond cut to collect detached grains and analyze grain size. The main length of grains was measured by using optical magnifiers (Leica, model: M165C, Leica Microsistemas S.L.U., Wetzlar, Germany) shown in Figure 2b. Then, the equivalent diameter was calculated by assuming the grain geometry as a sphere. The Anderson Darling test was applied to the diameters, and a *p*-value of 0.65 was obtained. Consequently, it could be assumed that the equivalent diameter had normal behavior, with an average value of 0.347 mm and confidence interval of 0.300–0.394 mm.

### 3.2. Power-Consumption Measurement

The plane dry grinding experiments were carried out in a grinding machine (KAIR, model: T650, KEHREN, Hennef, Germany) with a nominal power of 2.24 kW. The cutting conditions used during the experimental tests are listed in Table 1. These conditions are similar to the range of values used by Singh et al. [26]. Five grinding passes were made for each test specimen with dimensions of 30 mm × 10 mm × 130 mm. Material hardness was measured with a durometer (Wolpert, model: Testor HT, Buehler, Esslingen am Neckar, Germany). Table 1 shows the average error dispersion, with an interval of 95% confidence, of the material hardness of each metallic alloy. In total, five hardness values were recorded for each alloy. Then, an Anderson Darling test was applied to verify a normal distribution. The confidence interval was estimated by using a t-student test in the material’s hardness measurements.

**Figure 3:**
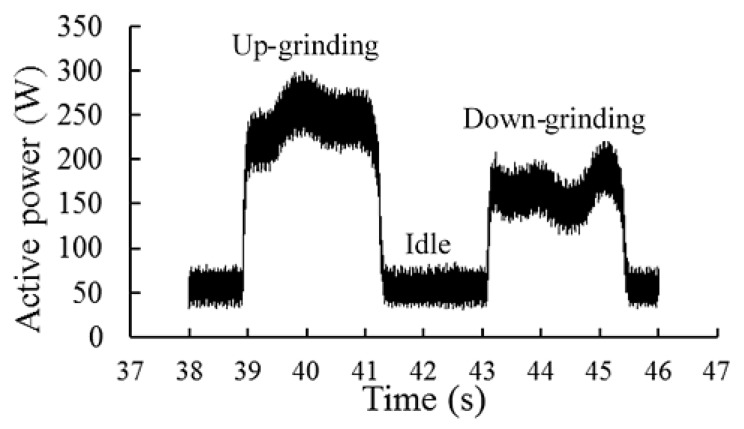
Signal of active power consumption by the electric motor.

To evaluate the power consumption, the active power of an electric motor was recorded in three conditions: idle, up-grinding, and down-grinding. Power was measured by an energy analyzer (HBM, model: Genesis eDrive Testing, Hottinger Baldwin Messtechnik GmbH, Darmstadt, Germany), where the current intensity, voltage, and the power consumed by the motor were recorded [27]. Since the grinding machine was a three-phase machine, a wattmeter recorded the measurements of the three phases. Then, these measurements were saved on files in ASCI format to be postprocessed. These results allowed to identify the tie periods and power consumption during cutting in up- and down-grinding, and an idle condition. Figure 3 shows the signals of consumed power while grinding C45K steel with a depth of cut of 0.020 mm and workpiece speed of 101 mm/s. In this figure, the path of up- and down-grinding and the idle condition were identified, with average active power consumption of 259, 240, and 54 W, respectively.

## 4. Results and Discussion

**Figure 4:**
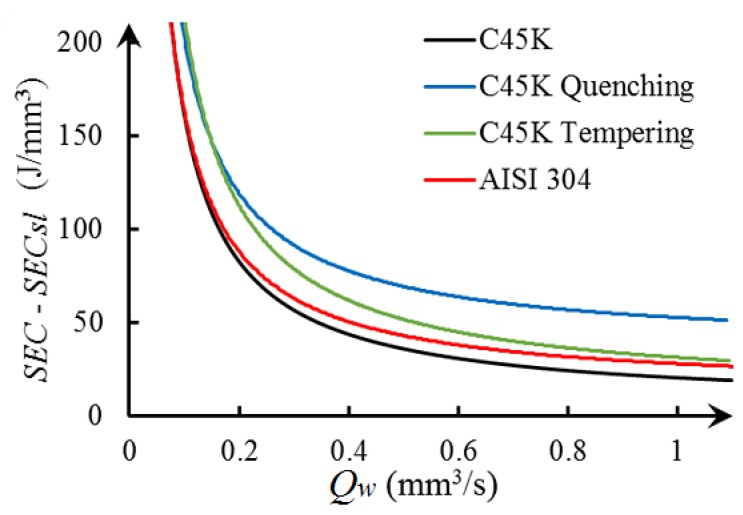
Specific energy consumption and material-removal rate.

In Equation (2), if terms *P*, $P_{sl}$, and $Q_{w}$ are known, then it is possible to find $P_{pl}$ and $SEC_{ch}$ by performing regression. These regression curves are estimated from experimental data obtained with different cutting conditions, such as depths of cut of 0.010, 0.015, and 0.020 mm; average workpiece speed of 57, 101, and 150 mm/s; and a constant peripheral cutting speed of 22.9 m/s. These cutting conditions are similar to those frequently used by other authors [26]. Figure 4 shows the regression curves of each material from the experimental data. In Equation (2), the specific energy consumed during grinding SEC is defined as the ratio between *P* and $Q_{w}$, while the specific energy consumed in sliding $SEC_{sl}$ is the ratio between the $P_{sl}$ and $Q_{w}$.

The regression applied to the experimental data of materials C45K, C45K quenching, C45K tempering, and AISI 304, have adjustment quality $R^{2}$ of 0.82, 0.84, 0.76, and 0.8, respectively. Figure 4 exhibits that, when $Q_{w}$ increases, SEC gradually decreases. From the graph, it is also noted that, if $Q_{w}$ is very small, then SEC is higher, which is defined as a size effect [15]. The quality of the adjustment allows to validate the hypothesis that SEC has asymptotic behavior defined by the Equation (2). This behavior is similar to the model developed by Diaz et al. [10] and by Zhong et al. [28] for milling and turning operations, respectively. Moreover, Table 2 shows tha material hardness of each metalic alloy and Table 3 exhibits the results of SEC associated with the mechanism involved in grinding, $SEC_{sl}$, $SEC_{pl}$, and $SEC_{ch}$, where the specific energy consumed by ploughing $SEC_{pl}$ is the ratio between $P_{pl}$ and $Q_{w}$. The average energy consumed by sliding $SEC_{sl}$ is 92%, 85%, 57%, and 94% of the total energy consumed by C45K, C45K quenching, C45K tempering, and AISI 304, respectively. This work characterizes the industrial-scale grinding process, where $SEC_{sl}$ is an order of magnitude greater than $SEC_{ch}$, as compared to other authors who studied grinding at the laboratory scale by using a single grain grinding wheel [6,26], which found small $SEC_{sl}$ values. In addition, the $SEC_{ch}$ values for C45K steel and C45K quenching reported in this work are similar in magnitude to the $SEC_{ch}$ values reported by Marinescu et al. [29]. Furthermore, $SEC_{sl}$, $SEC_{pl}$, and $SEC_{ch}$ of the C45K quenching steel present greater values than the other metallic alloys. This is due to the fact that the greater the material hardness of the workpiece is, the higher the required SEC for chip cutting is [10].

**Figure 5:**
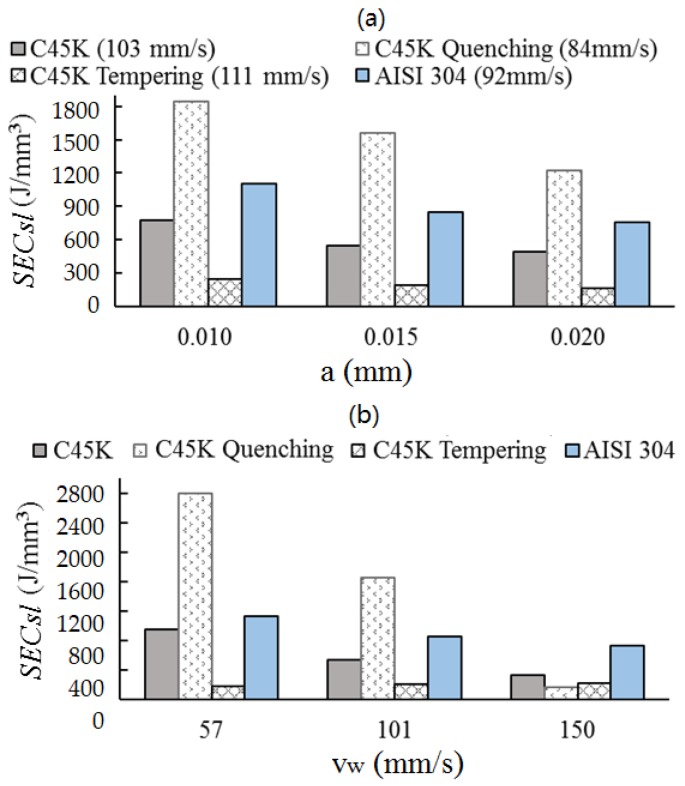
Relationship between specific energy consumption and (**a**) depth of cut, and (**b**) workpiece speed.

Figure 5a shows the results of $SEC_{sl}$ for different depths of cut, types of alloys, and thermal treatments. It is shown that the greater the depth of cut is, the lower the contribution of $SEC_{sl}$ is, which is similar to the behavior reported by Ghosh et al. [30]. This is due to the presence of a large number of cutting grains and, subsequently, the area subjected to friction is smaller [12]. Figure 5b exhibits the results of $SEC_{sl}$ for an average depth of 0.015 mm at different workpiece speeds and different materials. In general, $SEC_{sl}$ decreases as the speed of the workpiece increases. A similar behavior was reported by Bakkal et al. [23], who described that, in grinding, the ratio between tangential and normal forces increased as the speed of the workpiece increased.

The results show that high energy consumption is found for lower depths of cut and workpiece speeds, except for in the C45K tempering material, which shows constant values of energy consumption when these two operational parameters are increased. In particular, quenching requires more energy for low and medium speeds of the workpiece and depths of cut, whereas tempering presents similar low values of energy consumption for higher depths of cut and workpiece speeds. This can be due to differences in hardness at the surface of the materials and their elastoplastic behavior. Finally, both the C45K and AISI 304 materials exhibited the same trend of decreasing energy consumption by increasing depth of cut and workpiece speed. Thus, thermal treatments had a noticeable influence on energy consumption, but temperature in grinding is also crucial and depends on the selection of the operational parameters [31]. The model of material-removal rate $Q_{w}$ developed in the present study is different from other models, as the thickness of chip (7) and the section of cutting grain (8) are the function of the angular position of the grain. This is different from Zhenzhen et al. [16], who considered the maximum value of chip thickness to estimate material-removal rate $Q_{w}$. On the other hand, chip thickness (7) has the same variables and structure as defined by Malkin et al. [15]. The only difference is in the exponent that affects $C_{g}$ and the speed of the grinding wheel and workpiece. Other models calculated the material-removal rate as a product of depth of cut, grinding width, and workpiece speed [32]. This last model did not incorporate the speed of the grinding wheel in the definition of the material-removal rate as compared to the model presented in this work. Furthermore, this model stated the relationship between the three mechanisms in grinding. The energy consumed by ploughing depends on the material-removal rate that is, ultimately, associated with the selected cutting conditions. Thus, an increase of $Q_{w}$ produces a decrease of SEC–$SEC_{sl}$ and, also, a decrease of $P_{pl}$. This is not evident due to the nonlinear behavior of SEC–$SEC_{sl}$, as shown in Figure 4. On the other hand, $SEC_{ch}$ mainly depends on the material and is not sensitive to cutting conditions. Accordingly, SECch is constant and defined by the limit value of the asymptote. Furthermore, SECsl presents a linear trend, with an $R^{2}$ greater than 0.999 for cutting conditions *a* and $v_{w}$, as shown in Figure 5a,b. In particular, if *a* and $v_{w}$ increase, then, $Q_{w}$ and $P_{sl}$ also increase due to the linear behavior of $SEC_{sl}$ with respect to $Q_{w}$. Finally, sliding is the main mechanism of energy consumption in industrial-scale grinding. Therefore, an increase of $Q_{w}$ produces an increase in energy consumption in this process.

## 5. Conclusions

The present paper proposed a model to calculate material-removal rate $Q_{w}$ and specific energy consumption in grinding, where depth of cut, workpiece speed, effective cutting section, grain density, and material hardness play a crucial role. Accordingly, the main conclusions can be summarized as follows:

- A model was successfully developed to evaluate the dissipated energy by the sliding, ploughing, and chip-formation mechanisms in an industrial-scale grinding process. In general, sliding energy governs the process of energy dissipation in grinding.
- The dissipated energy by the sliding mechanism decreases when the depth of cut and workpiece speed increase, allowing to reduce energy consumption and manufacturing cost during grinding. The sliding mechanism represents, on average, 90% of the total energy consumed for the following materials: C45K, C45K quenching, and AISI 304.
- The model also allows to find the specific energy consumed by chip formation, which is the limit value defined by the asymptotic behavior experienced by SEC. This validates the hypothesis that, during down-grinding, the energy calculated by the analyzer corresponds to the energy dissipated by sliding.

For future work, we propose to study the relationship of the three mechanisms of sliding, ploughing, and chip formation when performing up- or down-grinding in industrial-scale grinding. Additionally, this study helps to optimize this process with the aim of reducing the energy consumption during up- or down-grinding operations. Accordingly, it is necessary to use a wider range of operational parameters $v_{w}$ and *a* to investigate SEC behavior and its local minimum.

## Figures and Tables

**Table 1:** Cutting conditions used during the experimental tests.

Cutting Parameter	Magnitude of Values		
Depth of cut (mm)	0.010	0.015	0.020
Peripheral cutting speed (m/s)	22.9	22.9	22.9
Speed of workpiece (mm/s)	57	101	150

**Table 2:** Material hardness of metallic alloys.

Hardness Material	C45K	C45K Quenching	C45K Tempering	AISI 304
(HRC)	17.35 ± 1.38	56.16 ± 0.52	25.72 ± 0.72	19.85 ± 0.68

**Table 3:** Average specific energy consumption of different indices in plane dry grinding.

Metallic Alloy	SEC (J/mm${}^{3})$	${\mathrm{SEC}}_{\mathrm{sl}}$ (J/mm${}^{3})$	${\mathrm{SEC}}_{\mathrm{pl}}$ (J/mm${}^{3})$	${\mathrm{SEC}}_{\mathrm{ch}}$ (J/mm${}^{3})$
C45K	655	602	30	8
C45K quenching	1805	1541	132	36
C45K tempering	351	201	113	11
AISI 304	958	901	36	13

## Nomenclature

θ	Angular position (°)
$\theta^{*}$	Dimensionless angular position
*a*	Depth of cut (mm)
$A_{cg}$	Section cut by a grain (mm${}^{2}$)
$b_{g}$	Width of grain during cutting (mm)
$C_{g}$	Grain number per unit area (mm${}^{-2}$)
$d_{g}$	Grain diameter (mm)
$D_{M}$	Diameter of the grinding wheel (mm)
*f*	Feed per grain (mm)
*h*	Undeformed chip thickness (mm)
*k*	Constant of proportionality
$l_{c}$	Contact length between wheel and workpiece (mm)
$l_{g}$	Distance between grains (mm)
$N_{g}$	Number of grains
*P*	Total power consumption (W)
$P_{ch}$	Power consumption by chip formation (W)
$P_{pl}$	Power consumption by ploughing (W)
$P_{sl}$	Power consumption by sliding (W)
$P_{v}$	Idle power consumption (W)
$Q_{w}$	Material removal rate (mm${}^{3}$/s)
$R_{g}$	Grain radius (mm)
$R_{M}$	Radius of the grinding wheel (mm)
SCE	Specific cutting energy (J/mm${}^{3}$)
SEC	Specific energy consumption (J/mm${}^{3}$)
$SEC_{ch}$	Specific energy consumed by chip formation (J/mm${}^{3}$)
$SEC_{pl}$	Specific energy consumed by ploughing (J/mm${}^{3}$)
$SEC_{sl}$	Specific energy consumed by sliding (J/mm${}^{3}$)
$v_{c}$	Peripheral cutting speed (m/s)
$v_{w}$	Speed of workpiece (m/s)
